# Prevalence and Risk Factors for Occupational Voice Disorders in Nepalese Teachers: A Cross-Sectional Study

**DOI:** 10.1055/s-0043-1777417

**Published:** 2024-04-12

**Authors:** Apar Pokharel, Puspa Basnet, Bibek Sharma, Hari Prasad Upadhyay

**Affiliations:** 1 Department of ENT and Head and Neck Surgery, College of Medical Sciences, Chitwan district, Province Number: 3, Nepal; 2 Department of Public Health, College of Medical Sciences, Chitwan district, Province Number: 3, Nepal; 3 Department of Community Medicine, College of Medical Sciences, Chitwan district, Province Number: 3, Nepal

**Keywords:** school teachers, voice disorder, voice fatigue

## Abstract

**Introduction** Teachers are a high-risk group for the development of vocal dysfunction, as they use voice extensively in their profession.

**Objective** To know the prevalence and risk factors associated with voice strain in teachers.

**Methods** A cross-sectional study was conducted among schoolteachers in Chitwan, Nepal. The Voice Handicap Index questionnaire was used as a survey tool.

**Result** A total of 315 teachers were enrolled in the study. The mean age of the participants was of 36.7 years. Teachers from public schools, primary grade classes, > 50 pupils in the classroom, > 24 hours of classes per week, dust in class, and recurrent tonsil problems were associated with various degrees of vocal handicap.

**Conclusion** There is a high prevalence of voice disorder among teachers. A holistic approach, which includes teacher education regarding voice care during their work and management of their voice handicap by taking into consideration different risk factors, must be adopted.

## Introduction

The primary tool for the performance of teachers in the classroom is their voice, which plays an important role in gaining respect, holding attention, and making work more interesting. The quality of the voice and the way teachers express lessons can positively influence the attention of the students to the subject matter. Any abnormality in the voice of teachers can negatively impact their communication skills, social life, personal emotions, and occupation. However, in the initial stages, symptoms related to dysphonia are often neglected, which further aggravates the problem. Delay in intervention makes recovery more challenging, which sometimes might result in quitting professional practice. 1

Menon et al. 2 conducted a study on professional voice disorder among teachers in Southern India, and they found that 45.4% of teachers were currently facing difficulties related to their voice, 52.8% had had some voice handicap in the previous year, and 70.1% had faced problems related to their voice during their teaching career. In 2004, Roy et al. 3 reported a higher prevalence of voice disorders among teachers (11%) compared to nonteachers (6.2%). Joshi et al. 4 performed 70° rigid laryngoscopy in 200 teachers who had voice handicap, and they found that 37 (18.5%) teachers had pathological findings in the larynx. A total of 70.3% of teachers in the pathological group were working in unfavorable environments (which had dust and poor acoustics, for example), and 64.9% were exposed to loud background noise (such as from traffic and neighboring classrooms). 4 A study 5 reported that voice problems accounted for one fourth of all occupational diseases in Poland; their prevalence increased from 1.9% in 1977 to 25% in 2001.

The risk factors for the development of dysphonia among teachers, such as the grade of the class, the number of years in the teaching profession, the subject matter, the number of weekly teaching hours, and the number of pupils in the class have all been studied. Delay in seeking medical help accounts for the persistence of problem among teachers. 3 6 7 8 9 Although there are numerous studies conducted in developed countries 6 7 8 , there is paucity of information in developing countries like Nepal. The main problems with developing countries like Nepal are poor school infrastructure, limited teaching aids like projectors, microphones etc., and a higher student-to-teacher ratio. 8 10 11 12 13

The main objective of the present study was to determine the self-reported prevalence of dysphonia among school teachers in Chitwan, Nepal, and to find individual lifestyle, work, and environmental risk factors for the development of voice problems. Through this study, we have also attempted to bridge the gap between the voice dysfunction of teachers and the medical team consisting mainly of otolaryngologists, voice therapists, and speech language pathologists.

## Methods

### Study Design and Participants

This questionnaire-based quantitative, field, observational, cross-sectional study was assessed and approved by the institutional Ethics in Research Committee (2019/18). Teachers aged between 18 and 61 years from 13 public and private schools in Chitwan were enrolled for the study from March to August, 2021. All staff actively engaged in teaching for > 1 year and teaching classes for at least 18 hours a week were enrolled. Patients with otolaryngological malignancies, and history of head and neck surgery and radiotherapy were excluded from the study. Similarly, patients currently on treatment with proton pump inhibitors or histamine type-2 (H2) receptor antagonists or type-1 antihistamines within a period of 4 weeks were also excluded.

### Instrument: Voice Handicap Index

The Voice Handicap Index (VHI) questionnaire was used as a survey tool ( Appendix 1 ). 14 The questionnaire addresses voice handicap in relation to vocal load and physical, environmental, and psychoemotional aspects. This patient-based self-assessment tool consists of 30 items, each with a score ranging from 0 to 4. These items are equally distributed throughout three domains: functional, physical, and emotional aspects of voice disorders. The functional subscale includes statements that describe the “impact of a person's voice disorders on his or her daily activities.” The emotional subscale indicates the patient's “affective responses to a voice disorder.” The items in the physical subscale include statements about the “self-perceptions of laryngeal discomfort and the voice output characteristics” of the patient. The overall aim of the VHI is to quantify the patient's perception of handicap because of their vocal function. The VHI questions include emotional, physical, and functional aspects. Each aspect includes 10 questions rated in a 5-point scale: never (0); almost never (1); sometimes (2); almost always (3); and always (4). The total score ranges from 0 to 120. Scores < than 30 indicate mild voice handicap, between 31 and 60, moderate voice handicap, and > 60, severe voice handicap. 14

The questionnaire ( Appendix 1 ) included information regarding teachers' age, gender, the years working in the teaching profession, the type of school (public/private), the type of class (primary/lower secondary/higher secondary), the number of students per class, and the weekly working hours. The questionnaire also comprised information regarding classroom work conditions, such as room temperature (< 18°C, 18°C to 21°C, or > 21°C), dust pollution (yes/no) and air conditioning (yes/no). Questions regarding smoking habits were also asked, as well as questions on the history of otolaryngological and general diseases, such as thyroid problems, nose and sinus diseases, allergies, and pharyngitis The frequency of vocal symptoms, such hoarseness, vocal fatigue, aphonia, feeling of dry throat, feeling of lump in the throat, and persistent dry cough, were also assessed, as well as data on the treatment for the voice handicap, such as use of medications, voice rehabilitation, and sick leaves from school due to voice disorders. 15

### Data Collection Procedures

Personal visits were made to different private and public schools in the Chitwan district. The principals of different schools were approached, and the aim of the present study was explained. They were asked to distribute the questionnaires among teachers. The principal estimated the number of questionnaires required for the school according to the number of teachers. The questionnaires were accompanied by instructions on how to fill them out. On the next day, the filled-out questionnaires were collected from the teachers at the school.

### Data Analysis

The data collected were tabulated in a spreadsheet and then analyzed by PASW Statistics for Windows (SPSS Inc., Chicago. IL, United States) software, version 18.0, with a significance level of *p* ≤ 0.05. Descriptive statistical analysis was used to express the variables in terms of mean, standard deviation (SD), frequency, and percentage values.

## Results

The mean age of the participants was of 36.7 (minimum: 18; maximum: 61) years. On average, the participants had been teaching for 9.2 (minimum: 1; maximum: 33) years. The results of the descriptive analysis of different continuous variables are provided in Table 1 .

**Table 1:** Results of the univariate analysis of continuous variables

S.N.	Variables	Mean	Standard deviation	Minimum	Median	Maximum
1	Age (in years)	36.7	10.4	18	35	61
2	Years of service	9.2	7.4	1	7	33
3	Number of pupils in class	56.9	26.1	18	45	130
4	Weekly class hours	27.1	11.6	6	29	72
5	VHI: hunctional score	10.0	7.4	0	9	31
6	VHI: physical score	9.5	7.2	0	9	29
7	VHI: emotional score	8.5	7.5	0	7	27
8	Total VHI score	27.9	21.6	0	25	85

Abbreviation: VHI, voice handicap index; S.N., serial number.

A total of 53% of the teachers female; however, no significant association between voice handicap and gender distribution was observed. We identified that 301 out of 315 (95.6%) participants had some kind of vocal handicap. The results of the descriptive analysis of grouped variables are provided in Table 2 .

**Table 2:** Results of the univariate analysis of grouped variables

S. N.	Variables	Number	Percentage
**1**	**Gender**		
	Female	167	53.0
	Male	148	47.0
**2**	**Type of school**		
	Public	132	41.9
	Private	183	58.1
**3**	**School grade**		
	Primary	109	34.6
	Lower secondary	86	27.3
	Higher secondary	120	38.1
**4**	**Number of pupils in class**		
	< 50	164	52.1
	≥ 50	151	47.9
**5**	**Years of service**		
	< 10	181	57.5
	10 to 20	107	33.9
	> 20	27	8.6
**6**	**Weekly class hours**		
	< 24	123	39.1
	≥ 24	192	60.9
**7**	**Subject taught**		
	All	27	8.6
	Computer science	11	3.5
	English	87	27.6
	Games	11	3.5
	Math	70	22.2
	Nepali	31	9.8
	Science	42	13.3
	Social studies	29	9.2
	Others (Accounting/Business/Drawing)	7	2.2
**8**	**Class temperature > 21°C**	239	75.9
**9**	**Dust in the classroom**	94	29.8
**10**	**Air conditioning**	98	31.1
**11**	**Speaking**		
	Low voice	60	19.1
	Raised voice	202	64.1
	Top of one's voice	53	16.8
**12**	**Physical exercise**	243	77.1
**13**	**Tobacco use**	10	3.2
**14**	**Allergy**	56	17.8
**15**	**Ear issues**	8	2.5
**16**	**Sinusitis**	34	10.8
**17**	**Tonsil issues**	80	25.4
**18**	**Thyroid issues**	12	3.8
**20**	**Phoniatric care**		
	History of pharmacotherapy for voice disorder	127	40.3
	History if voice rehabilitation	88	28
	History of sick leaves due to voice disorder	42	13.33

Abbreviations: S.N., serial number.

**Fig. 1:**
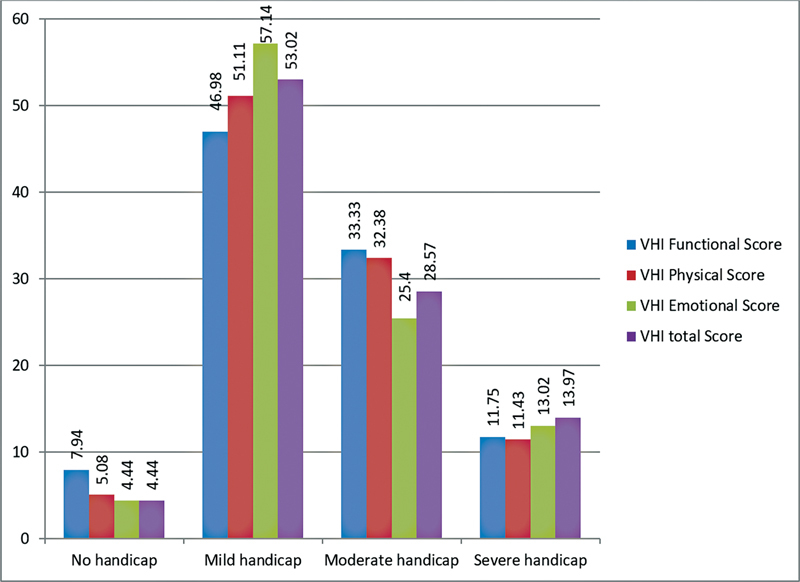
Distribution of severity of vocal symptoms, expressed in percentages.

**Fig. 2:**
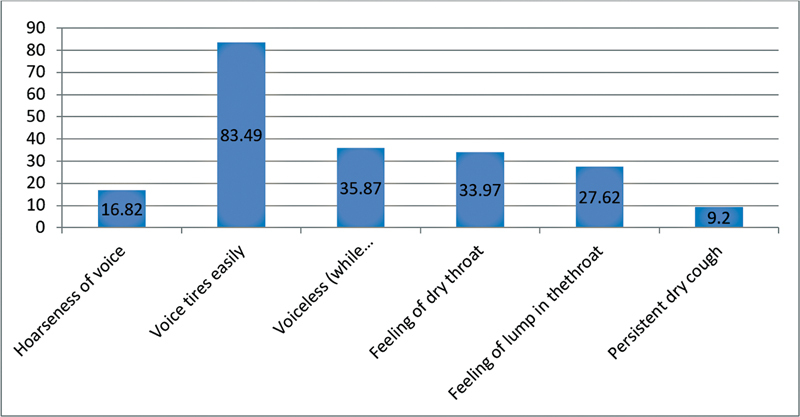
Frequency of vocal symptoms reported by teachers.

Out of the 315 teachers enrolled in the present study, only 14 did not complain of any symptoms, and most teachers had a mild grade of vocal problems. ( Figure 1 ) The most common vocal symptoms reported were vocal fatigue (83.49%), followed by aphonia and dry throat. ( Figure 2 )

In the bivariate analysis, the type of school ( *p =* 0.24), the grade (overall *p =* 0.13), the number of pupils in class ( *p =* 0.03), the years of service ( *p* < 0.01), dust in class ( *p =* 0.10), and tonsil issues ( *p =* 0.13) presented values of *p* ≤ 0.25, and, as per the relaxed *p* -value criteria, they could be used for the multivariate analysis ( Table 3 ). In the variance inflation factor (VIF) analysis, as all of these variables presented values < 2; thus, they were kept in the final multivariate model. In the multivariate analysis ( Table 3 ), primary grade teachers were identified to have significantly increased odds (adjusted odds ratio [AOR]: 18.4; 95% confidence interval [95%CI]: 1.5–221.0) of presenting a vocal handicap, while keeping other variables constant. Similarly, significantly increased odds (AOR: 2.8; 95%CI: 1.5–5.1) of presenting a vocal handicap were identified for the variable *years of service* when controlling for other variables such as type of school, grade, number of pupils in class, dust in class, and tonsillar issues. However, the presence of tonsillar issues was identified to significantly reduce the odds of being vocally handicapped (AOR: 0.2; 95%CI: 0.0–0.7] when controlling for other variables such as type of school, grade, number of pupils in class, years of service, and dust in class.

**Table 3:** Bivariate and multivariate logistic regression regarding vocal handicap (0 = no; 1 = yes)

S.N.	Variables	Odds ratio (95% confidence interval)	*p* -value	Adjusted odds ratio (95% confidence interval)	*p* -value
**1**	**Gender**				
	Female	Ref.			
	Male	0.9 (0.3–2.6)	0.82		
**2**	**Type of school**				
	Public	Ref.		Ref.	
	Private	1.9 (0.6–5.6)	**0.24**	3.6 (0.8–16.0)	0.09
**3**	**School grade**		**0.13 (overall)**		
	Primary	6.9 (0.9–55.5)	0.07	18.4 (1.5–221.0)	0.02*
	Lower secondary	2.1 (0.6–7.1)	0.22	2.3 (0.5–10.9)	0.28
	Higher secondary	Ref.		Ref.	
**4**	**Number of upils in class**	1.1 (1.0–1.1)	0.03*	1.0 (1.0–1.1)	0.32
**5**	**Years of service**	1.9 (1.3–2.9)	< 0.001*	2.8 (1.5–5.1)	< 0.001*
**6**	**Weekly class hours**	1.0 (0.9–1.0)	0.53		
**7**	**Subject taught**		0.93 (overall)		
		Ref.			
	Computer science	12100000 (0–Inf.)	0.99		
	English	0.4 (0.1–3.7)	0.45		
	Games	12100000 (0–Inf.)	0.99		
	Math	2.7 (0.2–44.0)	0.50		
	Nepali	0.6 (0.1–6.5)	0.64		
	Science	0.5 (0.1–5.1)	0.56		
	Social studies	12100000 (0–Inf.)	0.99		
	Others (Accounting/Business/Drawing)	12100000 (0–Inf.)	0.99		
**8**	**Class temperature**				
	≤ 21°C	1.9 (0.4–8.9)	0.39		
	> 21°C	Ref.			
**9**	**Dust in class**				
	No	Ref.		Ref.	
	Yes	0.4 (0.1–1.2)	**0.10**	0.7 (0.2–2.9)	0.65
**10**	**Air conditioning**				
	No	Ref.			
	Yes	0.6 (0.2–1.7)	0.34		
**11**	**Speaking**		0.29 (overall)		
	Low voice	Ref.			
	Raised voice	2.5 (0.8–8.3)	0.13		
	Top of one's voice	2.3 (0.4–12.5)	0.33		
**12**	**Physical exercise**				
	No	Ref.			
	Yes	0.9 (0.3–3.4)	0.89		
**13**	**Tobacco use**				
	No	Ref.			
	Yes	2050000 (0–Inf.)	0.99		
**14**	**Allergy**				
	No	Ref.			
	Yes	1.3 (0.3–6.0)	0.73		
**15**	**Ear issues**				
	No	Ref.			
	Yes	0.3 (0.0–2.7)	0.29		
**16**	**Sinusitis**				
	No	Ref.			
	Yes	0.7 (0.2–3.3)	0.67		
**17**	**Tonsil issues**				
	No	Ref.			
	Yes	0.4 (0.2–1.3)	**0.13**	0.2 (0.0–0.7)	0.02*
**18**	**Thyroid issues**				
	No	Ref.			
	Yes	2060000 (0–Inf.)	0.99		

Abbreviations: S.N., serial number.

Note: *(Relaxed *p* -value criteria considered in the univariate analysis.

## Discussion

Out of 315 teachers enrolled in the study, 301 had some kind of vocal handicap. The mean age of the participants was of 36.7 years, and 53% of the participants were female. The mean duration of the teaching practice was of 9.2 years, with a weekly average of 27.1 hours of classes. The mean number of pupils per class was of 57. In the regression analysis, we observed associations regarding public schools, primary grade classes, > 50 pupils in a classroom, > 20 years of service, > 24 hours of classes per week, dust in class, and recurrent tonsil problems and some degree of vocal handicap. Gender distribution and dust in the classroom ( *p* = 0.65) did not show any significant relationship with voice handicap. The average VHI score was of 27.9, and ∼40.3% of the teachers seek medical attention for the voice handicap. The most common vocal problem reported was vocal fatigue (83.49%).

We observed a higher prevalence of voice handicap due to voice disorders among the teachers of Chitwan than among European or American teachers, whose prevalence ranges from (11% to 32%) 3 7 8 15 The most probable explanation for this is the higher number of students in class, the longer years of service, and the higher number of weekly class hours in Nepal. A study conducted in Southern India showed that 72% of teachers had some voice handicap throughout their career. 2 In a study by Gotaas and Starr, 16 33.9% of the teachers seek medical help, a rate similar to that found in the current study. Preciado et al. 17 reported a higher prevalence of voice disorders in primary grade teachers, which is in line with the findings of the present study. Similar findings were also reported by Ubillos et al., 18 who reported that voice disorders were more common among primary grade teachers who taught in classrooms with large numbers of students. The most probable reason is increased noise production by small children, causing the teacher to speak more loudly, which could lead to voice handicap. Preciado et al. 17 also reported that the level of dust in the classroom is not a risk factor for the development of a voice handicap. Studies 2 4 17 19 have shown that teachers with > 20 years of experience were more likely to have a voice handicap, a finding similar to those of the current study. In terms of teaching load, many studies 20 21 22 have indicated that dysphonic teachers had longer weekly classroom hours than asymptomatic teachers. Many articles 8 23 24 in the literature also point out that voice handicaps among teachers are linked to the teaching of particular subjects, such as foreign languages, literature, mathematics, music, and physical education. However, in the present study, we did not find any significant correlation with the subjects taught. Devadas et al. 9 reported that the most common vocal symptom was vocal fatigue, and that 25% of the teachers consulted speech language pathologists for voice handicaps; among them, ∼75% reported improvement after speech therapy. Studies 5 25 have shown that 39% of Australian and 20% of American teachers have missed work due to a voice handicap. Upper respiratory tract infections, such as rhinitis, sinusitis, pharyngitis, and laryngitis were found to be significant risk factors among teachers who experience voice problems. 12 Studies 26 27 have also shown thyroid hormone disorder and issues pertaining to acid reflux are also risk factors for the development of voice disorders. However, in the present study, only recurrent tonsillitis showed a significant correlation with voice handicap among teachers.

There were some limitations to the present study. The cross-sectional design limited the evaluation of causality. As there was a lack of sufficient resources, comprehensive data on potential confounders and modifiers could not be obtained. A detailed clinical evaluation of the larynx, though flexible laryngoscopic and videostroboscopic examinations, was not conducted in the present study. Although smoking is a recognized cause of voice handicap, the possible contribution of smoking to the development of voice disorders among teachers could not be assessed in the present study, as only 3.2% of the teachers reported they were smokers. In addition, exposure to indoor air pollution secondary to cooking at home, teachers taking private tuitions and teachers shouting at their own children at home were not considered in the present study.

Teaching is a high-risk job for the development of voice handicaps. However, if adequate care is taken, the severity of the voice handicap can be controlled. Teachers must be provided with adequate training and education regarding voice optimization depending on background noise, number of students in class, number of weekly teaching hours, and room acoustics. They should also be made aware of different etiological factors, such as recurrent upper respiratory tract infections, thyroid disorders, and acid reflux disease, which could further worsen their vocal hygiene.

## Conclusion

Voice handicaps are very common. The more the voice organ loading factors, the greater the risk of developing a voice handicap. There are 98 public schools and 121 private schools in the Chitwan district. 28 However, no organized attempt has been made to collect data on the teachers' health issues in relation to their voice. This can lead to underdiagnosis of the problem and lack of timely intervention. The present study attempts to fill the gap and also stimulate the otolaryngological society of Nepal and the Ministry of Education to carry out longitudinal and prospective cohort studies to obtain more data on the severity of voice handicaps, their work-related determinants, and their consequences to the daily work performance of teachers.
