# Supplementary material for: Prevalence and Risk Factors for Occupational Voice Disorders in Nepalese Teachers: A Cross-Sectional Study
Source: Int Arch Otorhinolaryngol. 2024 Apr 12;28(3):e374–81. doi: 10.1055/s-0043-1777417 (PMC11226300; doi:10.1055/s-0043-1777417)
Supplement: Supplementary file 1 — Supplementary Material [file 10-1055-s-0043-1777417-s221311.pdf]

Appendix 1: Proforma with Voice Handicap Index Questionnaire

Questionnaire

Date:
Age/Sex:
Address of school:
School type: Government/Private
School level:
  Primary(less than class 5)
  Lower Secondary(6-8 class)
  Higher Secondary(9 class and above)

Pupils, number in attendance:
Years of employment:
Vocal effort, hours/week:
Use of chalk while teaching: Yes/No
Subjects taught:

Work and Life Hygiene

1. Ambient temperature of class:
   Below 18° C
   Between 18 and 21° C
   Above 21° C
2. Dustiness:
   Dust accumulation on curtains? Yes/No
   Blackboard wiping/chalk dust? Yes/No
3. Air agitation:
   Air conditioning? Yes/No
4. Phonation habits:
   Speaking in loud voice? Yes/No
   Speaking in a raised voice? Yes/No
   Speaking at the top of one's voice? Yes/No
5. Physical exercises/sport? Yes/No
   Regular
   Irregular
6. Tobacco use? Yes/No
   Years:
   Cigarettes per day:
7. Concomitant laryngological diseases:
   a) Sinusitis Yes/No
   b) Tonsil problem Yes/No
   c) Ear disease Yes/No
   d) Allergy Yes/No
   e) Thyroid disease Yes/No
   f) Decreased sleep Yes/No

Lifetime Vocal Symptoms

1. Hoarse voice
   chronic(permanent) Yes/No
   recurrent(periodical, less than 4 weeks) Yes/No
2. Voice tiredness? Yes/No
3. Voiceless while teaching? Yes/No
4. Dry throat? Yes/No

 © 2024. Fundação Otorrinolaringologia. All rights reserved.

5. Lump feeling in throat? Yes/No
6. Persistent dry cough? Yes/No

Phoniatric Care

1. Pharmacotherapy in the past? Yes/No
2. Voice rehabilitation in the past? Yes/No
3. Sick leave due to voice disorders in the past? Yes/No

VOICE HANDICAP INDEX
0-NEVER 1-ALMOST NEVER 2-SOMETIMES 3-ALMOST ALWAYS 4-ALWAYS

Part I-Functional

My voice makes it difficult for people to hear me.	0	1	2	3	4
People have difficulty understanding me in a noisy room.	0	1	2	3	4
My family has difficulty hearing me when I call them throughout the house.	0	1	2	3	4
I use the phone less often than I would like to.	0	1	2	3	4
I tend to avoid groups of people because of my voice.	0	1	2	3	4
I speak with friends, neighbors, or relatives less often because of my voice.	0	1	2	3	4
People ask me to repeat myself when speaking face-to-face.	0	1	2	3	4
My voice difficulties restrict my personal and social life.	0	1	2	3	4
I feel left out of conversations because of my voice.	0	1	2	3	4
My voice problem causes me to lose income.	0	1	2	3	4

SUBTOTAL:
Part II-Physical

I run out of air when I talk.	0	1	2	3	4
The sound of my voice varies throughout the day.	0	1	2	3	4
People ask, " What's wrong with your voice?"	0	1	2	3	4
My voice sounds creaky and dry.	0	1	2	3	4
I feel as though I have to strain to produce voice.	0	1	2	3	4
The clarity of my voice is unpredictable.	0	1	2	3	4
I try to change my voice to sound different.	0	1	2	3	4
I use a great deal of effort to speak.	0	1	2	3	4
My voice is worse in the evening.	0	1	2	3	4
My voice "gives out" on me in the middle of speaking.	0	1	2	3	4

SUBTOTAL:
Part III-Emotional

I am tense when talking to others because of my voice.	0	1	2	3	4
People seem irritated with my voice.	0	1	2	3	4
I find other people don't understand my voice problem.	0	1	2	3	4
My voice problem upsets me.	0	1	2	3	4
I am less outgoing because of my voice problem.	0	1	2	3	4
My voice makes me feels handicapped.	0	1	2	3	4
I feel annoyed when people ask me to repeat.	0	1	2	3	4
I feel embarrassed when people ask me to repeat.	0	1	2	3	4
My voice makes me feel incompetent.	0	1	2	3	4
I am ashamed of my voice problem.	0	1	2	3	4

SUBTOTAL:
TOTAL———————-

 © 2024. Fundação Otorrinolaringologia. All rights reserved.
